# Comprehensive evaluation of caloric restriction-induced changes in the metabolome profile of mice

**DOI:** 10.1186/s12986-022-00674-4

**Published:** 2022-06-27

**Authors:** Dadi Xie, Jinxi Huang, Qiang Zhang, Shiyuan Zhao, Hongjia Xue, Qing-Qing Yu, Zhuohao Sun, Jing Li, Xiumei Yang, Minglei Shao, Deshui Pang, Pei Jiang

**Affiliations:** 1 Department of Endocrinology, Tengzhou Central People’s Hospital, Xingtan Road, Tengzhou, 277500 China; 2 Department of General Surgery, The Affiliated Cancer Hospital of Zhengzhou University, Zhengzhou, China; 3 Clinical Laboratory, Tengzhou Central People’s Hospital, Tengzhou, 277500 China; 4 Jining First People’s Hospital, Jining Medical University, Jiankang Road, Jining, 272000 China; 5 Faculty of Science and Engineering, University of Nottingham Ningbo China, Ningbo, 315100 China; 6 Laboratory of Biochemistry and Biomedical Materials, College of Marine Life Sciences, Ocean University of China, Qingdao, 266003 China; 7 Department of Oncology, Jining First People’s Hospital, Jining, 272000 China

**Keywords:** Caloric restriction, Metabolite, Gas chromatography–mass spectrometry (GC–MS), Multivariate analysis, Lifespan

## Abstract

**Objects:**

Caloric restriction (CR) is known to extend lifespan and exert a protective effect on organs, and is thus a low-cost and easily implemented approach to the health maintenance. However, there have been no studies that have systematically evaluated the metabolic changes that occur in the main tissues affected by CR. This study aimed to explore the target tissues metabolomic profile in CR mice.

**Methods:**

Male C57BL/6J mice were randomly allocated to the CR group (n = 7) and control group (n = 7). A non-targeted gas chromatography–mass spectrometry approach and multivariate analysis were used to identify metabolites in the main tissues (serum, heart, liver, kidney, cortex, hippocampus, lung, muscle, and white adipose) in model of CR.

**Results:**

We identified 10 metabolites in the heart that showed differential abundance between the 2 groups, along with 9 in kidney, 6 in liver, 6 in lung, 6 in white adipose, 4 in hippocampus, 4 in serum, 3 in cortex, and 2 in muscle. The most significantly altered metabolites were amino acids (AAs) (glycine, aspartic acid, l-isoleucine, l-proline, l-aspartic acid, l-serine, l-hydroxyproline, l-alanine, l-valine, l-threonine, l-glutamic acid, and l-phenylalanine) and fatty acids (FAs) (palmitic acid, 1-monopalmitin, glycerol monostearate, docosahexaenoic acid, 16-octadecenoic acid, oleic acid, stearic acid, and hexanoic acid). These metabolites were associated with 7 different functional pathways related to the metabolism of AAs, lipids, and energy.

**Conclusion:**

Our results provide insight into the specific metabolic changes that are induced by CR and can serve as a reference for physiologic studies on how CR improves health and extends lifespan.

## Introduction

Caloric restriction (CR) is defined as a 20–40% reduction in caloric intake that is adequate for maintaining health without causing malnutrition [1]. There is increasing evidence that CR can slow aging and increase lifespan. In mice with 55–65% CR, mean lifespan and maximum lifespan were increased by 35–65% [2]. CR could inhibit the progression of the diseases, it has been shown to protect against aging-related diseases including cardiovascular and neurodegenerative diseases, type 2 diabetes (T2DM), and cancer [3–5]. The CR of physiological benefits on the body, including improved autophagy, modification of apoptosis, activation of cellular stress response, and alteration of hormonal balance [6]. However, the metabolic changes induced by CR that are responsible for these effects are poorly understood.

Gas chromatography–mass spectrometry (GC–MS), liquid chromatography or capillary electrophoresis coupled with MS (LC–MS/CE–MS), and nuclear magnetic resonance (NMR) are tools for high-throughput screening of biomarkers and metabolite profiling that are increasingly being applied to medical diagnostics [7]. Metabolomic analysis using these methods allows evaluation of changes in the relative abundance of low molecular weight metabolites such as amino acids (AAs), fatty acids (FAs), amines, organic acids, and nucleosides in physiologic and pathologic contexts. Among these methods, GC–MS can detect and quantify metabolites of molecular weight smaller than 650 Da [8]. There are other advantages in the GC–MS, such as higher chromatographic resolution and a larger database of identified peaks [9]. And there is a crucial procedure in GC–MS metabolomics analysis, which needs for the derivatization of the metabolite extracts into volatile and thermally stable derivatives [10]. Therefore, GC–MS being a highly sensitivity and high-throughput analytical platform, and has been widely used for untargeted analyses of primary metabolism [9].

In this study we investigated metabolic changes caused by CR by GC–MS-based high-throughput metabolomic profiling of biopsy samples from mice. We analyzed metabolite levels in tissues known to be affected by CR including serum, heart, liver, kidney, cortex, hippocampus, lung, muscle, and white adipose. Our results provide new insights into the physiologic effects of CR that can aid future research efforts to identify potential dietotherapy for diseases.

## Methods

### Diet and animals

Male C57BL/6J mice (9 weeks old) obtained from Jinan Pengyue (Jinan, China) were individually housed and allowed to acclimatize to the laboratory environment for 1 week. During this period, the mice had free access to water and a semipurified AIN-93M diet (XIETONG SHENGWU, Nanjing, China), and the amount of food consumed daily by each mouse was recorded. At 10 weeks of age, mice were randomly allocated to the CR or control group (n = 7 each). The latter had free access to food, whereas the CR mice were provided 50% of the calories consumed by the control group at 6 p.m. daily. The mice were maintained on the CR diet for 5 weeks with food intake and body weight recorded weekly. Experiments involving the mice were conducted according to the National Institutes of Health Guide for the Care and Use of Laboratory Animals and were approved by the ethics committee of Jining First People’s Hospital (protocol no. JNRM-2021-DW-005).

### Sample collection

After the CR period, mice were fasted overnight and euthanized with 1% sodium pentobarbital (50 mg/kg). Blood samples were collected from the cardiac coronary artery and separated by centrifugation (5000 × *g*, 5 min) to obtain serum. The heart, liver, kidney, cortex, hippocampus, lung, muscle, and white adipose tissue were dissected, weighed, and snap-frozen in liquid nitrogen and stored at − 80 °C until use.

### Sample preparation

A 100-μl volume of serum was mixed with 350 μl of methanol containing 100 μg/ml heptadecanoic acid (Sigma-Aldrich, St. Louis, MO, USA) as an internal standard; the mixture was vortexed and centrifuged at 14,000 × *g* for 10 min at 4 °C. The supernatant was transferred to a 2-ml tube and dried at 37 °C under a stream of nitrogen. An 80-μl volume of O-methyl hydroxylamine hydrochloride (J&K Scientific Industries, Ambala, India) dissolved in pyridine (Macklin Biochemical, Shanghai, China) was mixed with the extracts, followed by incubation for 90 min at 70 °C; 100 μl of N, O-bis(trimethylsilyl)trifluoroacetamide with 1% of trimethylchlorosilane (Sigma-Aldrich) was added to each sample, and the mixture was incubated at 70 °C for 60 min and analyzed by GC–MS.

Tissue samples (50 mg) were homogenized with 1 ml methanol (containing 1 mg/ml internal standard, Thermo Fisher Scientific, Waltham, MA, USA) and transferred to 2-ml tubes that were centrifuged at 20,913 × *g* for 10 min at 4 °C. The rest of the procedure was the same as for serum samples.

### Serum and GC–MS analysis

Triglycerides (TG) were measured in serum with kits from Wako Inc (Richmond, VA). Blood glucose was measured following a 4 h fast by tail-snip technique using handheld glucometer (ACCU-CHEK, Roche, IN). Serum urea levels were measured with QuantiChrom assay kit (BioAssay Systems, USA). Serum creatinine levels were determined with ELISA kit (Alpha diagnostic international, USA).

GC–MS analysis was performed on a Model 7890B gas chromatograph coupled to a 7000C mass spectrometer and equipped with HP-5MS fused silica capillary column (30 m × 0.25 mm × 0.25 μm; Agilent Technologies, Santa Clara, CA, USA). Each 1-μl aliquot of derivatized solution was run in split mode (50:1); the flow rate of helium gas through the column was 1 ml/min. The column temperature was held at 60 °C for 4 min, then increased by 8 °C/min to 300 °C and held at 300 °C for 5 min. The injection, transfer line, and ion source temperatures were 280 °C, 250 °C, and 230 °C, respectively. Twenty scans per second were recorded over the mass range of 50–800 m/z using electrospray ionization [11].

### Multivariate statistical analyses

MassHunter Unknowns Analysis and Quantitative Analysis (Agilent Technologies) were used to preprocess GC data, involving deconvolution, alignment, and data reduction to produce a list of m/z and RT pairs, with the corresponding intensities. The resulting table was exported into Excel and normalized. SIMCA-P v14.0 (Umetrics, Umea, Sweden) was used for statistical analyses. Orthogonal projection to latent structures discriminant analysis (OPLS-DA) was carried out to differentiate between the CR and control groups, with a variable importance in projection (VIP) value > 1.0 and *p* value < 0.05 considered significant. The CR group was further evaluated by permutation testing (200 permutations). SPSS v19.0 (SPSS, Chicago, IL, USA) was used for 2-tailed t tests, *p* value < 0.05 considered significant. MetaboAnalyst v5.0 (http://www.metaboanalyst.ca) and Kyoto Encyclopedia of Genes and Genomes (KEGG; http://www.kegg.jp) were used for functional analyses, and a raw *p* value < 0.5 and impact > 0 were defined as significant.

## Results

### Changes in body weight, blood glucose and TG induced by CR

**Fig. 1:**
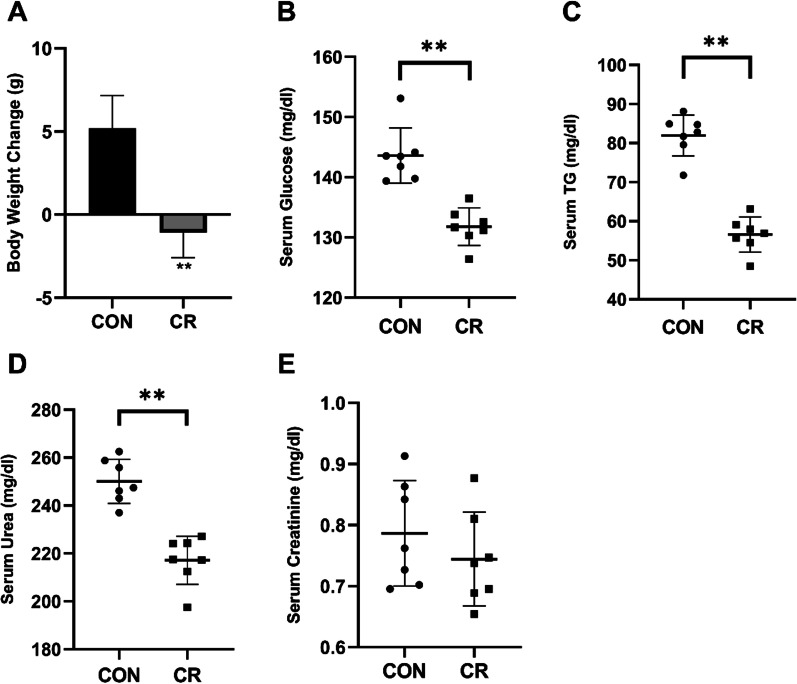
Effects of imposed CR on body weight, serum glucose, and serum TG. **A** Weight change in CR and control mice. **B** Serum glucose levels in CR group. **C** Serum TG levels in CR group. (D) Serum urea levels in CR group. **E** Serum creatinine levels in CR group. Values are means ± SD, n = 7, **p < 0.01 compared to the control group

At the begin of this study, the mice weighed 18.19 ± 0.98 g. Over the 35-day CR regimen, the weight of CR mice was lower than control mice (*p* < 0.01, Fig. 1A). Metabolic measurements showed that the CR groups had lower fasting blood glucose (*p* < 0.01, Fig. 1B), TG (*p* < 0.01, Fig. 1C) and urea (*p* < 0.01, Fig. 1D) levels compare to control group. There was no statistically significant change of plasma creatinine level between two groups (Fig. 1E).

### Metabolomic profiling of serum and tissue samples following CR

**Fig. 2:**
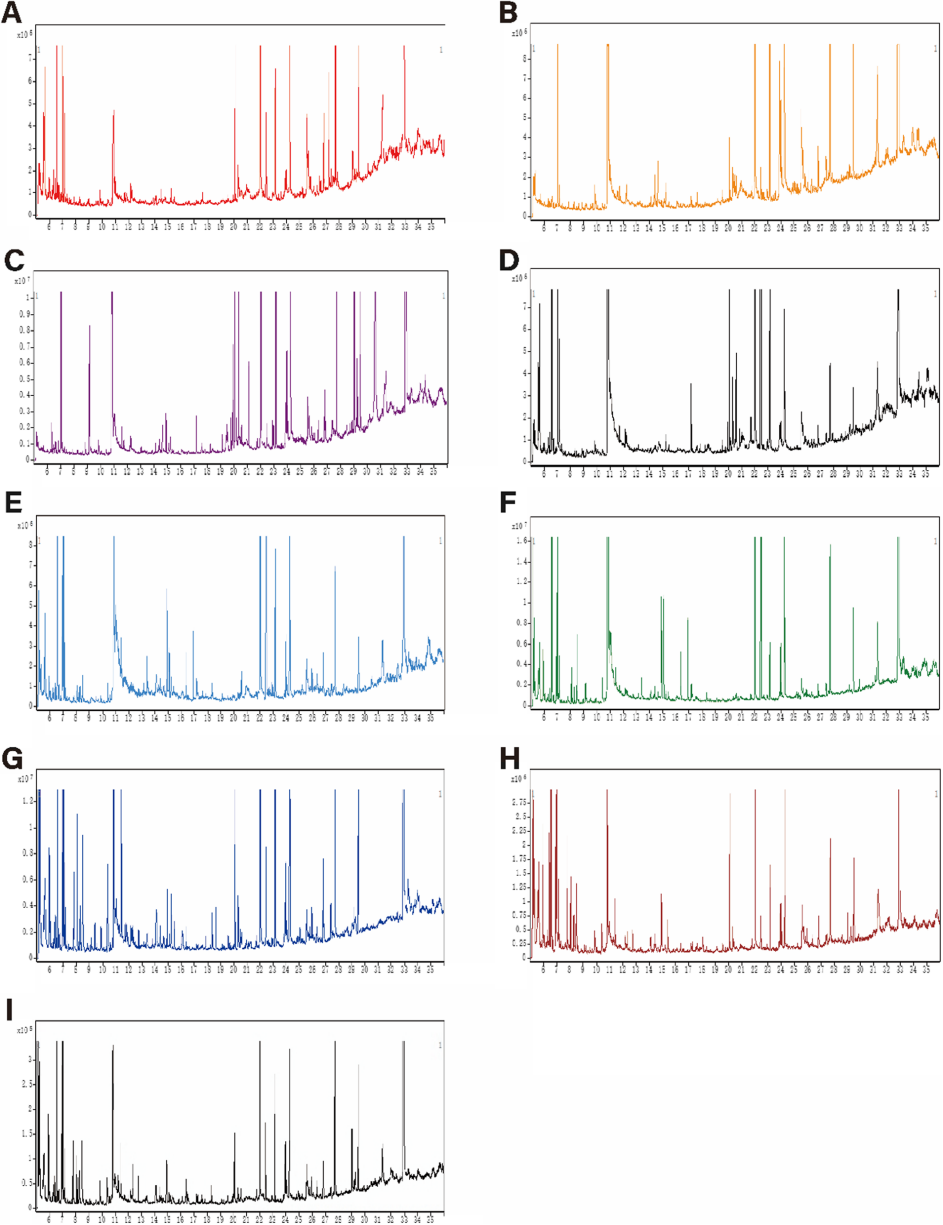
Representative GC–MS total ion current chromatograms from CR and control groups. **A** Serum, **B** heart, **C** liver, **D** kidney, **E** cortex, **F** hippocampus, **G** lung, **H** muscle, **I** white adipose

**Fig. 3:**
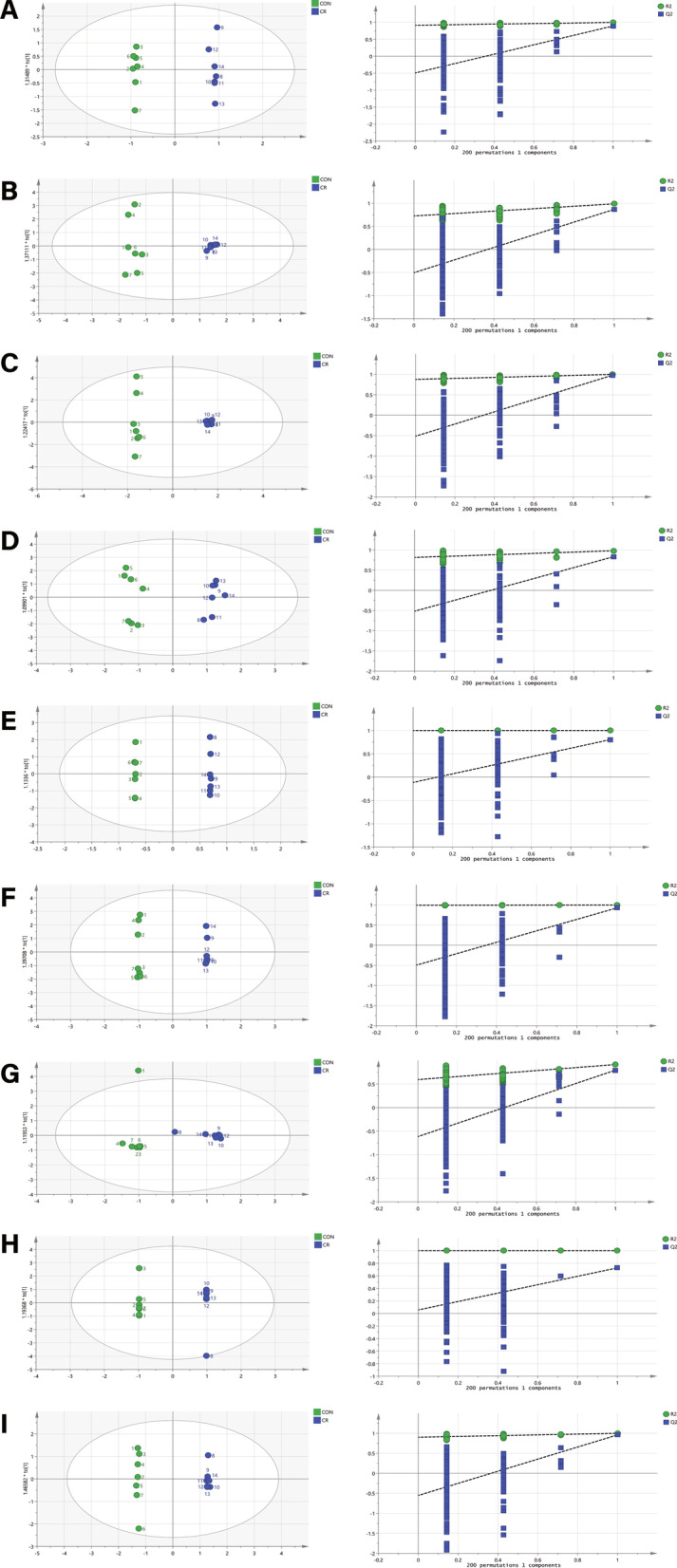
OPLS scores and 200 permutation tests for OPLS-DA models. **A** Serum, **B** heart, **C** liver, **D** kidney, **E** cortex, **F** hippocampus, **G** lung, **H** muscle, **I** white adipose. Statistical validation of the significant OPLS-DA models by permutation testing revealed no over-fitting (note that the blue regression line of the Q2 points intersect the vertical axis at values < 0)

Representative GC–MS total ion current chromatograms of the quality control serum and tissue samples (heart, liver, kidney, cortex, hippocampus, lung, muscle, and white adipose) from a mixture of CR and control mice all showed strong signals and good retention time reproducibility (Fig. 2). The OPLS-DA of the GC–MS data revealed a clear separation between the CR and control groups (serum: R2X = 0.497, R2Y = 0.998, Q2 = 0.891; heart: R2X = 0.684, R2Y = 0.988, Q2 = 0.866; liver: R2X = 0.833, R2Y = 0.997, Q2 = 0.979; kidney: R2X = 0.773, R2Y = 0.979, Q2 = 0.832; cortex: R2X = 0.859, R2Y = 1, Q2 = 0.803; hippocampus: R2X = 0.895, R2Y = 0.999, Q2 = 0.926; lung: R2X = 0.677, R2Y = 0.913, Q2 = 0.794; muscle: R2X = 0.88, R2Y = 1, Q2 = 0.727; and white adipose: R2X = 0.788, R2Y = 0.999, Q2 = 0.959); the values close to 1.0 indicated a stable model with predictive reliability. We identified 10 metabolites in the heart that showed differential abundance between the CR and control groups along with 9 in kidney, 6 in liver, 6 in lung, 6 in white adipose, 4 in hippocampus, 4 in serum, 3 in cortex, and 2 in muscle (VIP > 1, *p* < 0.05; Table 1). Statistical validation of the significant OPLS-DA models by permutation testing revealed no over-fitting (Fig. 3).

**Table 1:** Assigned metabolites detected in serum, heart, liver, kidney, cortex, hippocampus, lung, muscle, and white adipose at significantly differential levels between CR and control groups

Tissue	Metabolite	VIP	*P*	FC
Serum	Palmitic acid	1.21	0.00	0.69
	l-Valine	2.28	0.04	0.07
	l-Proline	2.80	0.00	0.13
	l-Alanine	1.20	0.03	0.79
Heart	l-Alanine	1.57	0.01	0.00
	Hexanoic acid	1.32	0.01	0.17
	l-Valine	1.52	0.00	0.06
	l-Isoleucine	1.49	0.01	0.04
	l-Threonine	1.49	0.02	0.01
	l-Aspartic acid	1.49	0.01	0.05
	l-Glutamic acid	1.58	0.01	0.00
	l-Phenylalanine	1.31	0.03	0.10
	d-Myo-Inositol	1.25	0.00	0.24
	Adenosine	1.26	0.03	0.10
Liver	Glycine	1.47	0.03	0.01
	Butanedioic acid	1.02	0.02	2.51
	l-Aspartic acid	1.28	0.03	0.13
	d-Mannose	1.08	0.02	0.31
	d-Myo-Inositol	1.14	0.01	0.28
	Doconexent	1.07	0.00	0.46
Kidney	3-Hydroxybutyric acid	1.34	0.00	0.51
	l-Serine	1.62	0.01	0.23
	Glycerol	2.06	0.00	0.18
	2-Butenedioic acid	1.20	0.00	0.57
	l-Aspartic acid	1.76	0.00	0.23
	l-Hydroxyproline	1.45	0.02	0.30
	d-Arabinose	1.72	0.00	0.33
	Oleic acid	1.34	0.00	0.49
	Stearic acid	1.01	0.01	0.64
Cortex	Glycine	2.72	0.03	5.46
	Glycerol	1.39	0.01	0.68
	Phosphoric acid	1.82	0.03	2.45
Hippocampus	l-Isoleucine	1.70	0.04	0.23
	l-Proline	1.87	0.03	0.14
	l-Aspartic acid	1.50	0.03	0.46
	Octadecanamide	1.32	0.00	0.54
Lung	Propanoic acid	2.10	0.00	9.30
	Morpholine	1.55	0.00	2.17
	Aminomalonic acid	1.84	0.00	5.77
	5-Oxoproline	2.10	0.00	14.50
	Hexadecanamide	1.78	0.03	0.07
	9-Octadecenamide	2.06	0.00	0.06
Muscle	l-Proline	1.64	0.02	2.54
	l-Aspartic acid	1.97	0.04	0.20
White adipose	Glycine	1.61	0.00	0.10
	Palmitic acid	1.17	0.00	0.44
	Myo-Inositol	1.12	0.00	0.48
	1-Monopalmitin	1.12	0.00	0.40
	Glycerol monostearate	1.19	0.01	0.32
	Cholesterol	1.36	0.00	0.28

FC, fold change (caloric restriction to control); VIP, variable importance in projection

**Fig. 4:**
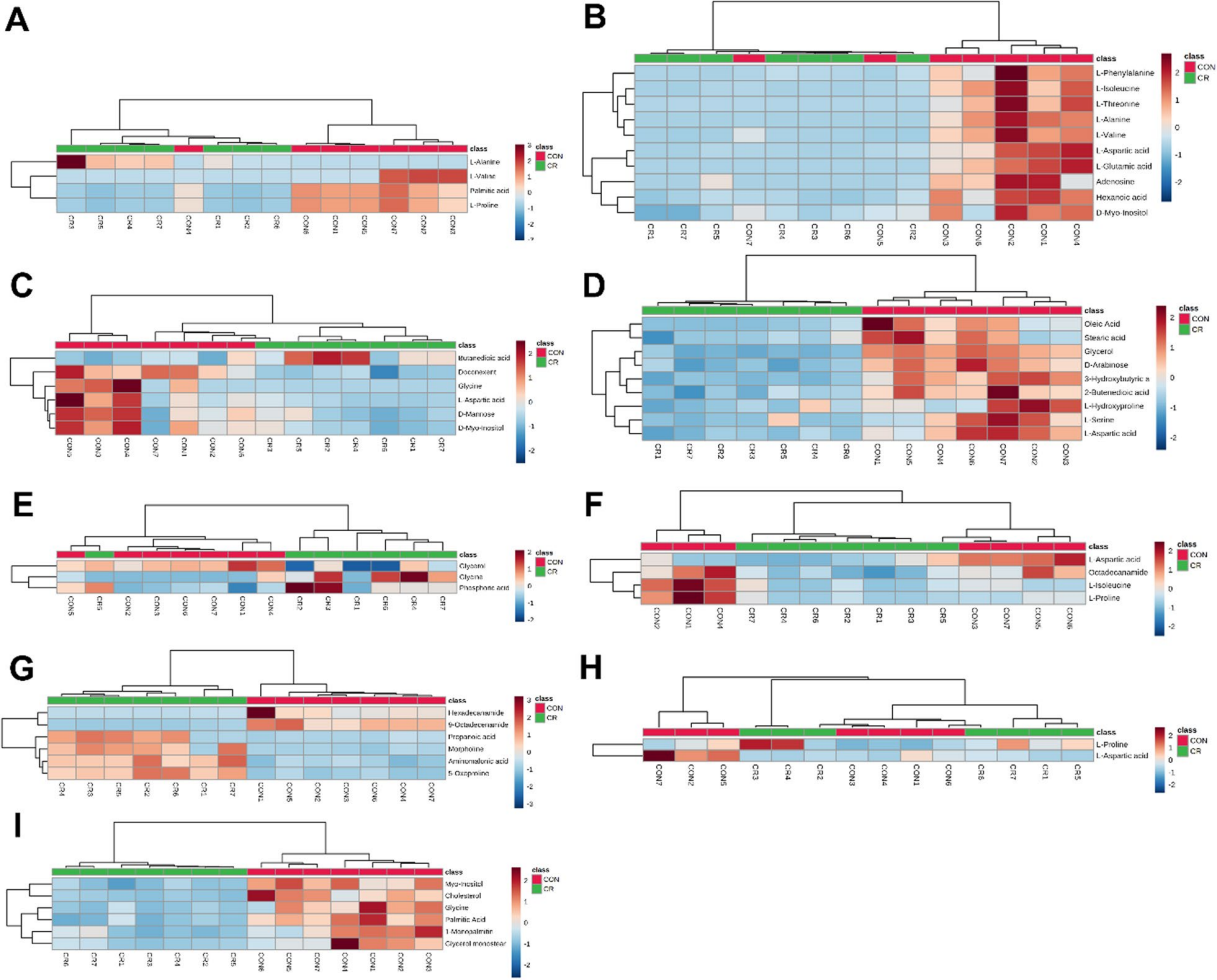
Heatmap of differentially expressed metabolites in CR and control groups. **A** Serum, **B** heart, **C** liver, **D** kidney, **E** cortex, **F** hippocampus, **G** lung, **H** muscle, **I** white adipose. Red and blue represent upregulation and downregulation, respectively; the shade is proportional to the degree of change. Rows and columns correspond to samples and metabolites, respectively

To further evaluate the differences in metabolism between the CR and control groups, we analyzed the data for the identified metabolites using MetaboAnalyst v5.0. Consistent with the results of the OPLS-DA, most samples were clearly grouped into 2 distinct clusters that showed minimal overlap (Fig. 4).

### Metabolic pathways affected by CR

**Fig. 5:**
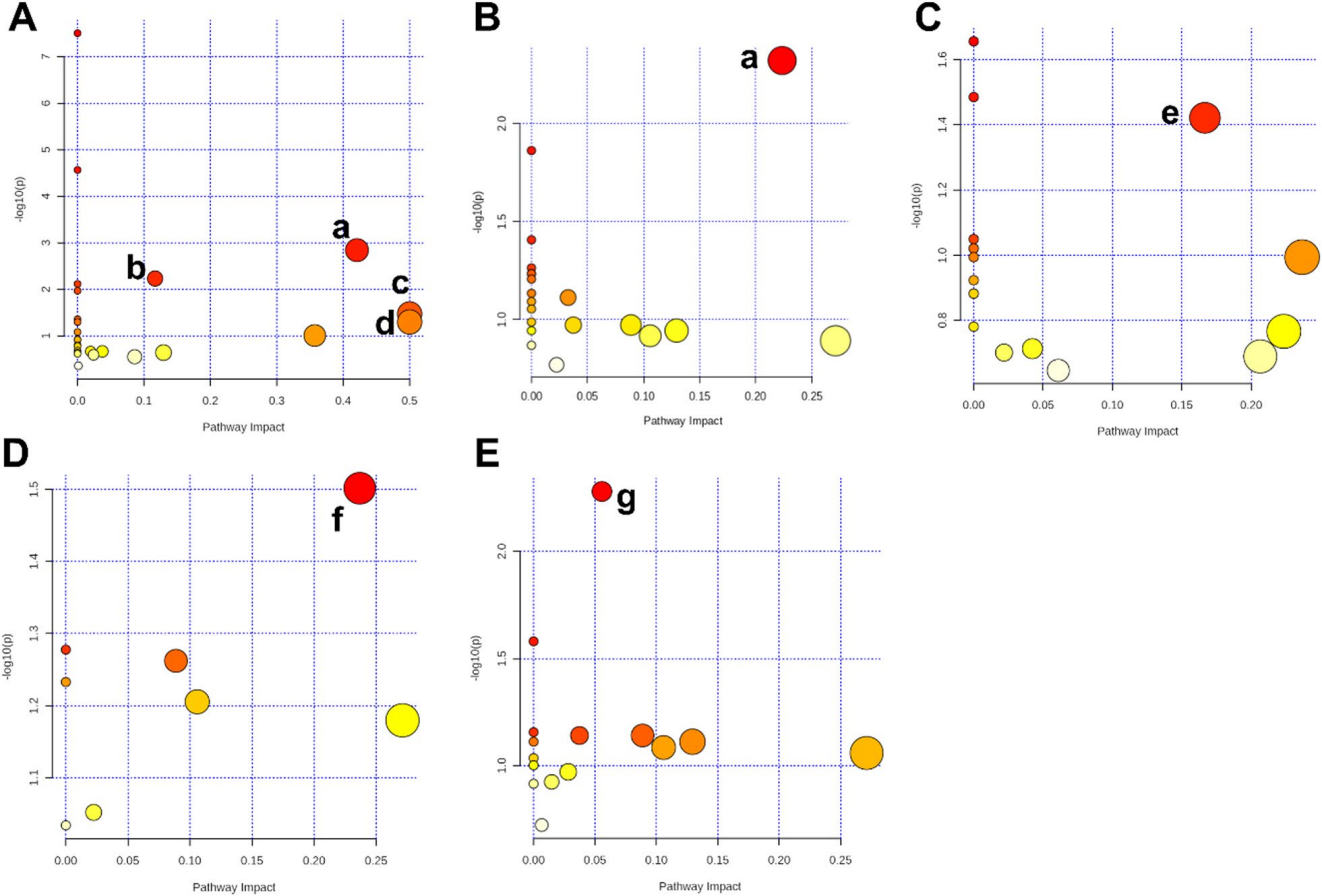
Summary of pathway analysis using MetaboAnalyst v5.0. **A** Heart, **B** liver, **C** kidney, **D** cortex, **E** white adipose. (a) Alanine, aspartate, and glutamate metabolism. (b) Arginine biosynthesis. (c) Phenylalanine, tyrosine, and tryptophan biosynthesis. (d) d-Glutamine and d-glutamate metabolism. (e) Aminoacyl-tRNA biosynthesis. (f) Glycerolipid metabolism. (g) Primary bile acid biosynthesis

**Fig. 6:**
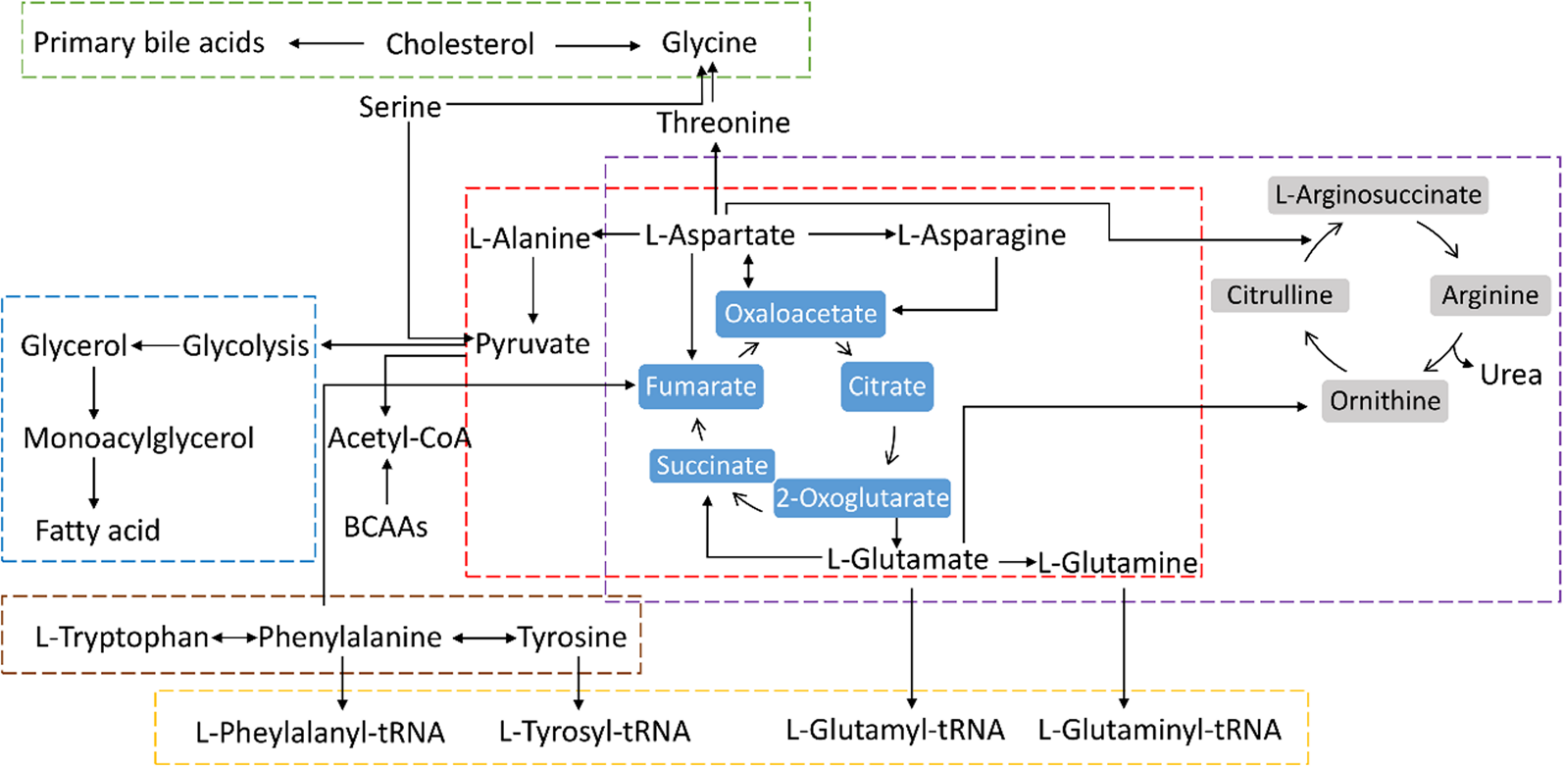
The diagram of metabolites and metabolic pathways in the main tissues under CR condition (red dotted line: alanine, aspartate and glutamate metabolism; purple dotted line: arginine biosynthesis; brown dotted line: phenylalanine, tyrosine and tryptophan biosynthesis; green dotted line: primary bile acid biosynthesis; blue dotted line: glycerolipid metabolism; yellow dotted line: aminoacyl-tRNA biosynthesis)

Integrated pathway analysis was performed to identify the metabolic pathways affected by CR. There were several significant KEGG pathways (raw *p* < 0.5, impact > 0; Table 2) including alanine, aspartate and glutamate metabolism, arginine biosynthesis, phenylalanine, tyrosine, and tryptophan biosynthesis, and d-glutamine and d-glutamate metabolism in the heart; alanine, aspartate, and glutamate metabolism in the liver; aminoacyl-tRNA biosynthesis in the kidney; glycerolipid metabolism in the cortex; and primary bile acid biosynthesis in white adipose. The impact value of the altered metabolic pathways ranged from 0.06–0.5. The results of the pathway analysis are provided in Table 2, with highlights shown in Fig. 5. A summary of metabolites and metabolic pathways is shown in Fig. 6.

**Table 2:** Results of the pathway analysis using MetaboAnalyst v5.0

Tissue	Pathway	Raw *p*	Holm adjust	FDR	Impact
Heart	Alanine, aspartate and glutamate metabolism	6.34E−04	5.20E−02	4.05E−02	0.42
	Arginine biosynthesis	3.46E−03	2.80E−01	7.27E−02	0.12
	Phenylalanine, tyrosine and tryptophan biosynthesis	2.63E−02	1.00E+00	2.84E−01	0.5
	d-Glutamine and d-glutamate metabolism	3.92E−02	1.00E+00	3.30E−01	0.5
Liver	Alanine, aspartate and glutamate metabolism	6.61E−03	5.55E−01	5.55E−01	0.22
Kidney	Aminoacyl-tRNA biosynthesis	2.46E−02	1.00E+00	7.37E−01	0.17
Brain	Glycerolipid metabolism	3.15E−02	1.00E+00	9.26E−01	0.24
White adipose	Primary bile acid biosynthesis	5.25E−03	4.41E−01	4.41E−01	0.06

*FDR* false discovery rate

## Discussion

Metabolomic approaches are used to detect and analyze changes in the abundance of low molecular weight metabolites in biological samples [12]. CR diets will inevitably lead to significant changes in the nutrition supply and the turnover of substances in vivo. The metabolism of the whole body is involved in an issue of dynamic equilibrium. When the food intake declined, the nutrition mainly comes from adipose and muscle tissue to meet the demand of body for nutrition. As expected, at baseline and after the intervention we observed significant correlations of circulating metabolites with CR. Here we used GC–MS and multivariate statistical analysis to examine the metabolic profile of mice following CR with the aim of identifying metabolites that are associated with the protective effects of CR and may reveal the underlying physiologic mechanisms. CR improved markers of general health and body weight and reduced plasma lipid, fasting glucose, and urea (Fig. 1A–D). Previous study had reported that CR reduced by 26% of epididymal fat mass of mice compared with control group, which showed CR could cause rapid weight loss, reduced whole-body fat mass [13]. However, plasma creatinine level was not affected significantly by CR diet (Fig. 1E).

The GC–MS analysis revealed 35 metabolites showing differential abundance between the CR and control groups; several AAs, FAs, and metabolites related to the tricarboxylic acid cycle and urea cycle were present significant change in CR mice (Table 1). AAs participate in many metabolic pathways as well as protein synthesis and are an important energy source. The AAs that showed differential abundance between the 2 groups were glycine, l-isoleucine, l-proline, l-aspartic acid, l-serine, l-hydroxyproline, l-alanine, l-valine, l-threonine, l-glutamic acid, and l-phenylalanine. Consistent with these results, the KEGG functional pathways enriched under CR included alanine, aspartate, and glutamate metabolism; phenylalanine, tyrosine, and tryptophan biosynthesis; arginine biosynthesis.

In this study, the main metabolite change is amino acids, which implied dietary protein may be the key determinant in CR on metabolism. We found that the level of all most AA were decreased in the CR group in our study. CR diet with high protein cannot extend lifespan, mice fed a low protein diet lived longer than high protein diet [14]. Individuals in a high protein diet have a higher incidence of developing diabetes and cardiovascular mortality than a low protein diet [15, 16]. Randomized controlled trials indicated that humans fed a protein restriction diet can improve metabolic health, decrease cancer incidence and mortality rates in individuals under 65 [17]. CR delays aging through protein restriction act on cellular pathways, including AMPK, mTOR, and GH/insulin/IGF-1 pathways [18]. Dietary protein and AA can influence the level of insulin, IGF-1 and activity of mTOR [14, 19, 20]. Protein restriction acts on these pathways reduces anabolic responses, increases mitochondrial function and autophagy, and thus delays aging [18]. Mammalian target of rapamycin (mTOR) is a master regulator in cell metabolism that senses cellular energy status and integrates signals from nutrients, growth factors, and stress factors to promote cell growth [21]. Restriction of dietary protein protects the important organ and promotes longevity through inhibition of mTOR pathway, improves the stress resistance after hepatic injury and protects from renal ischemia reperfusion injury [22, 23]. Study found that reduced dietary protein inhibits tumor growth and also inhibits mTOR activity in tumor [24]. Chronic overnutrition increases mTOR signaling, which is associated with the development of insulin resistance (IR) [25]. CR in humans is able to increase insulin sensitivity, which is beneficial to health and longevity [26]. In fasting condition, the utilization of energy diverted from cellular growth to protection, protection against oxidative stress by the repression of translation [27]. mTOR regulates translation of mRNA by phosphorylating the translation inhibitors, promotes translation initiation of mRNA, thus decreasing mRNA translation by mTOR inhibition that slows aging [28]. Therefore, in our study the reduction of AA content may be associated with decreased mRNA translation.

We also observed the level of l-isoleucine and l-valine were decreased compared to the control group in our study. Branched chain amino acids (BCAAs) may be an important role in AA regulation of metabolic health. The BCAA catabolism in human mostly in muscle, adipose and liver. BCAT and BCKDH are the two important gene in the BCAA metabolic pathway, which may affect the changes of BCAA in plasma [29]. Restricting the level of BCAAs in low protein diet can improve the mice metabolic health as same as low protein diet [20]. Amino acids can regulate IR, and reduction in fasting AA concentrations could improve insulin sensitivity effectively. Study showed that IR is associated with elevated concentrations of BCAAs and related metabolites [30]. The level of BCAAs was found to be increased in obesity, IR, T2DM, coronary disease (CAD) and metabolic syndrome [31]. Alternation of the level in BCAAs could serve as biomarker for the metabolic syndrome. BCAAs was also found to be able to activate mTOR activity in cell culture, rodents and humans [32–35]. BCAAs restriction lower mTOR activity to improve metabolic health and longevity [17]. So, reduction of BCAAs in diet can improve IR and metabolic health by restricting mTOR activity. Moreover, BCAAs could exacerbate mitochondrial dysfunction by increasing acylcarnitine accumulation in muscle [36]. In heart failure, the accumulation of BCAA and its metabolites may cause oxidative stress and impair mitochondrial function [37, 38]. Thus, CR protects tissues against oxidative stress damage by reduction of BCAAs.

In addition, aromatic amino acids, phenylalanine and tryptophan are elevated in individuals with obesity and IR [39, 40]. Our study showed that the level of phenylalanine is significantly decreased in the CR group, which may be used as a biomarker for T2DM developing. Glutamic acid is used to synthesize glutamine, and the l-glutamine content regulates the mTOR pathway, preventing cellular uptake of l-glutamine leads to inhibition of mTOR signaling and activation of autophagy [41].

CR also provides a protective effect against obesity. Randomized clinical trial showed that non-obese adults had a significant weight loss thorough decreased visceral fat under CR diet [42]. Adipose tissue is the primary site for the synthesis of FAs, which are the main energy source under fasting conditions, TG stored in adipocytes hydrolyzed to fatty acids and release ATP after oxidation. In our study, 4 FA metabolites were significantly decreased in white adipose tissue of the CR group compared to control mice including palmitic acid, 1-monopalmitin (MG [16:0/0:0/0:0]), glycerol monostearate (MG [0:0/18:0/0:0]), and cholesterol. The levels of docosahexaenoic acid, 16-octadecenoic acid, oleic acid, stearic acid, and hexanoic acid were also altered by CR, which was associated with changes in glycerolipid metabolism and primary bile acid biosynthesis.

Elevated cholesterol is a main risk factor that induces the development of atherosclerosis, long-term CR results in beneficial effects on cardiovascular disease by decreasing cholesterol [43]. CR has been shown to increase the expression of genes involved in FA oxidation and decrease that of genes related to FA synthesis [44–47]. In obesity and T2DM, enhanced of adipose lipolysis cause the increasing of glycerol and fatty acids, and inflammatory cytokines, and lead to IR [48, 49]. CR exerts a potent anti-inflammatory effect, reduce inflammation and improve human health [50]. Furthermore, excessive FA could impair mitochondrial function of muscle and cause incomplete β-oxidation [51]. Our study showed that the levels of palmitic acid, 1-monopalmitin, and glycerol monostearate—which are markers of endogenous FA synthesis—were decreased in the adipose tissue of CR mice. Thus, the regulation of FA metabolism may be a health benefit of CR, which was supported by our finding that FA synthesis was decreased along with FA oxidation in different tissues of CR mice. CR may also reduce oxidative damage; FA oxidation increases the flavin adenine dinucleotide (FADH)/nicotinamide adenine dinucleotide (NADH) ratio, which suppresses reactive oxygen species production [52]. CR was shown to protect the heart by inhibiting cardiac remodeling and fibrosis and increasing contractility and energy generation via lipid β-oxidation in mice [53]. In a clinical trial, FA was more effectively mobilized and oxidized in the fasting state induced by CR [54]. The pathway of primary bile acid biosynthesis showed a significant change. Bile acids emulsify dietary fats, activate lipase, catalyze the hydrolysis of glycerol into fatty acids, and promote the absorption of these lipids [55]. Moreover, bile acids stimulate enterocyte secretion of gut hormones, glucagon-like peptide 1 (GLP-1), which augments insulin secretion to help maintain glucose homeostasis [56].

## Conclusion

In conclusion, we established GC–MS and multivariate statistical analysis for metabolomic analysis of tissue samples to investigate the metabolic changes induced by CR. We found that CR altered the levels of various metabolites in multiple tissues, and especially in change of AA and FA metabolism. The long-term CR could be benefit to body, especially the reduction of protein has effect on the prolongation of lifespan. However, the effect of short-term CR on metabolism could not exclude stress and other factors. In the further, we will design this study at several time points, instead of one time point, which help to understand the changes in metabolites. Also, additional studies are needed in order to elucidate the specific molecular mechanisms associated with these effects and to determine whether these findings are applicable to humans. Nonetheless, our results provide a basis for future investigations on how CR can be used to increase longevity and improve health.

## Data Availability

The datasets used and analyzed during the current study are available from the corresponding author on reasonable request.

## Abbreviations

AAs: Amino acids
BCAAs: Branched chain amino acids
CAD: Coronary disease
CR: Caloric restriction
FADH: Flavin adenine dinucleotide
FAs: Fatty acids
GC–MS: Gas chromatography–mass spectrometry
GLP-1: Glucagon-like peptide 1
IR: Insulin resistance
LC–MS/CE–MS: Liquid chromatography or capillary electrophoresis coupled with MS
mTOR: Mammalian target of rapamycin
NADH: Nicotinamide adenine dinucleotide
NMR: Nuclear magnetic resonance
OPLS-DA: Orthogonal projection to latent structures discriminant analysis
TG: Triglycerides
T2DM: Type 2 diabetes
VIP: Variable importance in projection
